# Severe Corneal Endothelial Cell Loss Following FLACS: A Case Report

**DOI:** 10.3390/jcm15218465

**Published:** 2026-10-30

**Authors:** Robert H. Eibl

**Affiliations:** Independent Researcher, 67435 Neustadt, Germany; robert.eibl@alumni.dkfz.de

**Keywords:** FLACS, endothelial cell density, corneal edema, DMEK, case report

## Abstract

Femtosecond laser-assisted cataract surgery (FLACS) may reduce ultrasound energy and is often considered favorable for corneal endothelial safety, but clinically relevant endothelial injury can still occur. We report a 60-year-old man with normal preoperative endothelial cell density (ECD) who developed marked corneal edema and Descemet’s folds on the first day after apparently uncomplicated FLACS. No early postoperative specular microscopy was performed, and an intraocular lens (IOL) exchange was subsequently carried out while corneal clarity remained compromised. Several months later, a tertiary center documented an ECD of 743 cells/mm^2^ and subsequently 634 cells/mm^2^ in the affected eye, corresponding to a 71–75% reduction from the preoperative value of 2560 cells/mm^2^, with marked pleomorphism and polymegathism. Marked corneal edema and Descemet’s folds on postoperative day 1 documented clinically significant endothelial dysfunction before the subsequent IOL exchange; however, because ECD was not measured between the two procedures, the final magnitude of endothelial cell loss cannot be attributed exclusively to the initial surgery. Endothelial keratoplasty was discussed, but during approximately two years of follow-up the ECD remained markedly reduced, while the cornea remained clinically stable, and keratoplasty has not been required. This case emphasizes early endothelial assessment when persistent corneal edema or delayed visual recovery follows cataract surgery, particularly before further intraocular intervention.

## 1. Introduction

Femtosecond laser–assisted cataract surgery (FLACS) has been increasingly used as an alternative to standard phacoemulsification [1]. Potential advantages include greater capsulotomy precision, improved lens fragmentation, and enhanced refractive predictability. Some institutions promote FLACS as a means to reduce intraoperative ultrasound energy, with the aim of improving corneal endothelial safety. Although several studies have reported comparable or slightly reduced endothelial cell loss with FLACS, significant interindividual variability exists, and clinically important endothelial decompensation remains possible [2,3,4]. Early recognition of endothelial compromise appears particularly important when postoperative corneal edema persists.

The corneal endothelium is a nonregenerating monolayer that maintains stromal deturgescence and corneal transparency. After endothelial injury, surviving cells enlarge and migrate to cover the posterior corneal surface, producing increased cell-size variability and loss of hexagonality. In healthy adults, endothelial cell density (ECD) is commonly around 2500 cells/mm^2^, whereas the ability to maintain corneal clarity becomes increasingly limited as ECD approaches a critical range of approximately 400–500 cells/mm^2^. Cataract surgery itself causes some endothelial cell loss, but reported mean losses in otherwise healthy corneas are generally in the single-digit to low-teen percentage range rather than the magnitude observed in the present case [5,6].

Early postoperative corneal edema is therefore clinically relevant not only as a transient optical problem but also as a possible marker of endothelial injury. In a prospective study of phacoemulsification, the degree of corneal swelling on the first postoperative day correlated strongly with later permanent endothelial cell loss [7]. At the population level, FLACS does not appear to cause greater endothelial injury than conventional phacoemulsification; a 2023 meta-analysis of 42 trials found lower early endothelial cell-loss percentages after FLACS at several postoperative time points and no significant difference in ECD at most follow-up intervals [8]. A postoperative endothelial loss of more than 70% is therefore far outside the average changes reported in comparative studies and warrants analysis of the perioperative course.

This report describes a patient with normal baseline endothelial status who developed unexpected, severe endothelial cell loss after seemingly uncomplicated FLACS, followed by an IOL exchange performed without prior endothelial evaluation. The case is presented in detail to examine the timing of the endothelial dysfunction, relevant differential diagnoses and possible mechanisms, the implications for re-intervention in an eye with persistent postoperative edema, and the longer-term course of a cornea with markedly reduced endothelial reserve.

## 2. Case Presentation

### 2.1. Patient Information

A 60-year-old man presented with a visually significant age-related nuclear cataract in his right eye and an early, asymptomatic cataract in the left eye (Table 1). He had no history of corneal disease, prior ocular surgery, or corneal endothelial dysfunction. Family history was negative for corneal dystrophies. Preoperative examination revealed a clear cornea with normal endothelium, normal intraocular pressure, and unremarkable posterior segment findings. Preoperative specular microscopy CEM-530; NIDEK CO., LTD., Gamagori, Aichi, Japan) showed normal ECD for age (right: 2560 cells/mm^2^; left: 2907 cells/mm^2^). Preoperative Scheimpflug tomography (Pentacam; OCULUS Optikgeräte GmbH, Wetzlar, Germany) showed a minimum corneal thickness of 608 µm in the right eye and 591 µm in the left eye. The operating institution promoted routine FLACS as offering enhanced precision and improved endothelial safety. Extended-depth-of-focus (EDOF) IOLs were discussed and confirmed preoperatively as the preferred option.

### 2.2. FLACS Procedure

FLACS of the right eye was performed on 3 September 2024. According to the operative report, the laser patient interface was connected to the eye, parameters for capsulotomy, the main incision, paracenteses, and lens position and depth were set, and the femtosecond laser was applied for approximately 2 min before the docking system was released. Subsequent lens removal was performed using a Centurion (Alcon, Fort Worth, TX, USA) phacoemulsification system. The operative report described a hard nucleus and additionally noted “vis a tergo”. Viscoelastic material was used during the procedure, followed by phacoemulsification of the remaining nuclear material, cortical aspiration, implantation of the IOL, aspiration of the viscoelastic material, hydration of the incisions, and intracameral cefuroxime. The operative documentation also recorded topical/gel and corneal surface anesthesia as well as 1 mL intracameral 1% lidocaine. No intraoperative complication was recorded. The available operative documentation did not specify the femtosecond laser platform, patient-interface type, number of docking attempts, cumulative dissipated energy or comparable ultrasound-energy parameters, detailed fluidic settings, or the specific ophthalmic viscosurgical device used. The patient recalled unusually intense pressure during docking, followed by retraction and re-docking of the apparatus. He also recalled that preoperative ocular imaging had to be repeated twice on the day of operation because the initial images were reportedly unavailable in the operating room. According to his recollection, an iodine-containing ocular disinfectant had already been applied before this repeat imaging, which was followed by unusually prolonged persistence of afterimages corresponding to a regular pattern of multiple bright points. He further recalled a comment by the surgeon suggesting a transient shortage of fluid during aspiration. These observations were not documented as complications in the operative report and their relationship to the subsequent endothelial injury cannot be established retrospectively. A monofocal Clareon™ Toric IOL (Alcon, Fort Worth, TX, USA; +16.5 D, CYL 1.0; axis 98°) was implanted instead of the preoperatively discussed EDOF lens. Standard postoperative medication was prescribed.

### 2.3. Early Postoperative Course

On postoperative day 1, the patient reported marked vision loss (“seeing only fog”). Examination by the local ophthalmologist documented massive corneal edema with prominent Descemet’s folds and decimal visual acuity of 0.1. The patient also recalled that, during intraocular pressure measurement on postoperative day 1, he was unable to bring the green central fixation target (green dot) of the device into view despite attempting to align the operated eye. The assistant performing the measurement noted this difficulty and indicated that the measurement could nevertheless be obtained without fixation. Intraocular pressure on postoperative day 1 was measured four times, yielding 22, 24, 26, and 25 mmHg in the right eye (mean 24.3 mmHg) and 20, 20, 20, and 19 mmHg in the left eye (mean 19.8 mmHg). At postoperative day 13, corresponding measurements were 24, 24, 22, and 24 mmHg in the right eye (mean 23.5 mmHg) and 21, 21, 21, and 22 mmHg in the left eye (mean 21.3 mmHg). No pressure-lowering treatment was initiated. The patient recalled that by postoperative day 13 he was again able to perceive the green central fixation target with the operated right eye. The local ophthalmologist expressed concern regarding possible endothelial injury and advised delaying surgery on the fellow eye. A self-photograph obtained on postoperative day 3 showed a focal arcuate area of conjunctival hyperemia adjacent to the limbus (Supplementary Figure S1). The origin of this finding and any relationship to the laser patient interface cannot be established retrospectively. At postoperative days 6 and 13, edema had only mildly improved, and visual acuity remained reduced (0.5). No specular microscopy was performed. Over subsequent weeks, visual recovery remained slow and incomplete. Because of the patient’s repeated complaints regarding the unexpected postoperative visual outcome, the institution re-evaluated its records and documented that the implanted IOL was not the preoperatively discussed EDOF model. Repeat measurements were obtained, and an IOL exchange was scheduled.

At day 36, Descemet’s folds were still present and decimal visual acuity was 0.63 (subjectively 0.8). The unoperated left eye remained markedly myopic (−6.25 D with −0.5 D cylinder), resulting in substantial anisometropia. The IOL exchange was undertaken on day 41, approximately six weeks after the initial surgery, while corneal recovery was still incomplete. No endothelial cell count had been obtained before this second intraocular procedure.

### 2.4. IOL Exchange

The IOL exchange was performed on 14 October 2024, 41 days after the initial surgery, while corneal recovery remained incomplete. Persistent Descemet’s folds and reduced visual acuity had still been documented on day 36, five days before the exchange. No endothelial cell count had been obtained between the initial procedure and this second intraocular intervention. The available documentation for the exchange consisted of a brief procedural record confirming explantation of the previously implanted IOL and implantation of an AcrySof™ IQ Vivity™ Toric IOL (+16.5 D, CYL 1.0; axis 98°). No detailed operative report describing the explantation technique, extent of intraocular manipulation, capsular or zonular findings, use of an ophthalmic viscosurgical device, or other procedural parameters was included in the medical records available to the author. Consequently, the potential additional mechanical or endothelial stress associated with the IOL exchange cannot be reconstructed in detail. On the first postoperative day after the exchange, decimal visual acuity was 0.3. Temporal corneal edema, moderate Descemet’s folds, and 2+ anterior chamber cells were documented, and the fundus was not visible. By postoperative day 4, the corneal edema had resolved and only mild Descemet’s folds remained. By day 49 after the initial surgery, visual acuity had improved to 0.63 and the IOL was documented as stable, although subjective haze and subsequently symptomatic floaters persisted.

### 2.5. Surgery on the Fellow Eye

The left eye later underwent FLACS with implantation of an AcrySof™ IQ Vivity™ EDOF IOL (+15.5 D, non-toric). A brief postoperative elevation in intraocular pressure occurred but resolved with diuretic treatment.

The early postoperative course of the fellow eye was notable for a transient marked rise in intraocular pressure and a brief episode of dark-green visual obscuration, both of which resolved with treatment. A posterior capsular rupture in the left eye was identified later at another tertiary center and was retrospectively considered likely to have occurred intraoperatively, although no such complication had been documented at the time of surgery. Despite these findings, the fellow eye retained substantially higher endothelial cell density and relatively preserved endothelial morphology during subsequent follow-up.

### 2.6. Second Tertiary Center Evaluation

Several months later, the patient sought an independent evaluation at a second tertiary center. Specular microscopy using the CEM-530 (NIDEK CO., LTD., Gamagori, Aichi, Japan) showed a right-eye ECD of 743 cells/mm^2^ (71.0% loss from the preoperative value of 2560 cells/mm^2^; Table 2) with marked pleomorphism and polymegathism, whereas the left eye showed 2309 cells/mm^2^ (20.6% loss) with relatively preserved morphology. At a subsequent examination on day 330, specular microscopy showed an ECD of 634 cells/mm^2^ in the right eye (75.2% loss from baseline) and 2304 cells/mm^2^ in the left eye (20.7% loss), with marked pleomorphism and polymegathism in the right eye and relatively preserved endothelial morphology in the left eye (Figure 1; Table 2). At the day-330 examination, the central ECD value of 634 cells/mm^2^ was derived from 15 identified cells in that individual measurement field. The same multipoint CEM-530 examination included four additional sampled locations, with 56 endothelial cells identified across the five fields in total. Site-specific ECD values ranged from 634 to 957 cells/mm^2^. Repeated multipoint measurements were available at subsequent examinations on 5 November 2025 and 9 June 2026 (Table 2). Thus, the central value of 634 cells/mm^2^ should be interpreted as a local estimate rather than as a precise global endothelial cell density. The marked reduction relative to the preoperative ECD was nevertheless supported by the additional sampled locations and by subsequent multipoint examinations.

The early postoperative corneal edema and Descemet’s folds suggested that substantial endothelial dysfunction had already occurred during or shortly after the initial surgical episode. However, because specular microscopy was not performed before the subsequent IOL exchange, an additional contribution of the second intraocular procedure to the final endothelial cell loss cannot be excluded. DMEK was recommended as a possible future treatment should progressive corneal decompensation develop.

Values are given in cells/mm^2^. Percentage endothelial cell loss was calculated from the central/single-site value as [(preoperative ECD-postoperative ECD)/preoperative ECD] × 100. ECD: endothelial cell density.

### 2.7. Follow-Up

As shown in Table 2, serial measurements demonstrated persistently and markedly reduced ECD in the right eye, with substantial regional variation across measured corneal locations. The central/single-site value increased from 634 cells/mm^2^ in July 2025 to 968 cells/mm^2^ in November 2025 and 1046 cells/mm^2^ in June 2026. However, concurrent multipoint measurements also showed considerable spatial variation, ranging from 691 to 968 cells/mm^2^ in November 2025 and from 728 to 1046 cells/mm^2^ in June 2026. These apparent numerical increases should therefore not be interpreted as endothelial regeneration, but are more consistent with regional heterogeneity, sampling variability, and redistribution and enlargement of surviving endothelial cells. The clinically relevant finding was the persistence of a markedly reduced endothelial reserve compared with the preoperative ECD of 2560 cells/mm^2^. The fellow eye also showed regional variation on serial multipoint measurements, while central ECD values remained relatively similar at 2304, 2332, and 2381 cells/mm^2^ in July 2025, November 2025, and June 2026, respectively. Clinical stability in this report refers to maintenance of corneal transparency and visual function and should not be interpreted as numerical stability of ECD, which showed substantial regional and inter-examination variability. The patient initially reported worsening morning vision, compatible with reduced endothelial reserve. Fine posterior capsular opacification of the right eye had already been documented at day 260 and became visually significant during later follow-up.

Despite the markedly reduced ECD, the cornea remained clinically stable and no endothelial keratoplasty has been required to date. Posterior capsular opacification was treated by Nd:YAG laser capsulotomy. On the following day, visual acuity was 1.0 in both eyes. Symptoms of ocular surface dryness are treated with topical hyaluronate eye drops.

### 2.8. Patient Perspective

As the patient in this report, I experienced the prolonged visual impairment and uncertainty regarding the cause of the postoperative corneal edema as particularly distressing. Surgery of the fellow eye, originally planned only a few days later, had to be postponed for approximately two months, resulting in a major interocular refractive difference of about 6.5 diopters during this period. The later documentation of severe endothelial cell loss helped explain the prolonged recovery. Despite the markedly reduced endothelial cell density, subsequent stabilization and the recovery of good visual acuity after Nd:YAG capsulotomy have so far allowed endothelial keratoplasty to be avoided.

## 3. Discussion

This case demonstrates that severe endothelial injury can occur after FLACS even in an eye with normal preoperative ECD. Although FLACS may reduce ultrasound energy, it does not eliminate risks associated with docking, fluidics, laser-related effects, or mechanical trauma. Comparative studies generally show similar endothelial outcomes, with some meta-analyses reporting lower early endothelial cell loss after FLACS [1,4,8]. The discrepancy between the patient-reported perioperative events and the operative record, which documented no complication, cannot be resolved retrospectively.

The magnitude of endothelial cell loss distinguishes this case from routine postoperative endothelial change. Published reviews describe mean endothelial losses after phacoemulsification in healthy eyes in an approximate range of 1.8–15%, while recent pooled FLACS data generally show endothelial outcomes comparable with or somewhat better than those after conventional phacoemulsification [5,6,8]. The documented reduction from 2560 cells/mm^2^ preoperatively to 743 cells/mm^2^ and later 634 cells/mm^2^ therefore cannot be explained as an expected postoperative decrease. The marked pleomorphism and polymegathism are also consistent with compensatory enlargement and redistribution of surviving endothelial cells after substantial cell loss [5,6].

Rare severe corneal complications after FLACS have nevertheless been reported. Nosé et al. described four cases of Descemet membrane detachment (DMD) associated with femtosecond-laser corneal incisions; in one case, delayed recognition was followed by persistent corneal edema and ultimately required DMEK. Chen et al. similarly reported severe DMD after FLACS that was initially mistaken for transient endothelial swelling and was subsequently confirmed by anterior-segment OCT. In a larger retrospective series of 510 FLACS eyes, laser-associated DMD was identified in 3.9%, with pre-existing corneal guttata and ECD below 2000 cells/mm^2^ as significant risk factors. These reports concern identifiable Descemet membrane complications rather than isolated endothelial cell loss and therefore are not directly comparable with the present case, but they illustrate that severe postoperative corneal edema after FLACS may occasionally reflect significant structural or endothelial injury and warrants early diagnostic assessment [9,10,11].

Persistent corneal edema immediately after cataract surgery has a broad differential diagnosis. Relevant possibilities include direct endothelial trauma, excessive ultrasound or fluidic stress, Descemet membrane injury or detachment, toxic anterior segment syndrome, retained material, postoperative inflammation, elevated intraocular pressure, and decompensation of previously unrecognized endothelial disease [5,12]. In the present case, the normal preoperative ECD, clear cornea, negative family history for corneal dystrophy, and relatively preserved endothelial morphology in the fellow eye argue against a pre-existing bilateral endothelial dystrophy. On postoperative day 1, the mean IOP was 24.3 mmHg in the affected eye compared with 19.8 mmHg in the fellow eye; this modest pressure elevation alone would not readily explain the severity of the corneal edema and prominent Descemet’s folds. No infection was documented. Early specular microscopy, pachymetry, and anterior-segment optical coherence tomography were not performed, and no quantitative grading of anterior chamber inflammation was available after the initial FLACS. Preoperative Pentacam tomography documented minimum corneal thicknesses of 608 µm in the right eye and 591 µm in the left eye; at the day-330 endothelial examination, corneal thickness recorded by the specular microscope was 655 µm in the right eye and 626 µm in the left eye. Because these measurements were obtained with different instruments and do not necessarily represent identical corneal locations, direct quantitative comparison is limited. Therefore, a transient or localized Descemet membrane injury, toxic or inflammatory endothelial insult, or other intraoperative endothelial stress cannot be reconstructed or excluded retrospectively. The available findings support substantial postoperative endothelial dysfunction as the clinical syndrome without establishing a single mechanism.

Among the possible perioperative contributors, the patient recalled unusually intense pressure during docking, followed by retraction and re-docking of the laser interface. These observations were not documented as intraoperative complications and should therefore be interpreted cautiously. Transient intraocular pressure elevations during femtosecond-laser docking are well documented and vary with the patient-interface system [13,14]; however, there is no evidence that docking caused the endothelial injury in this case. The absence of information on the laser platform, patient-interface type, and number of docking attempts further limits mechanistic interpretation. Phacoemulsification energy, fluidic stress, laser-related effects, and other mechanical or toxic endothelial insults remain alternative or potentially contributing factors.

The timing of the IOL exchange is clinically important. Marked edema and Descemet’s folds were already present on postoperative day 1 and had not fully resolved by day 36, indicating reduced endothelial reserve before the second operation. In eyes with endothelial dysfunction, low ECD, increased corneal thickness, and morning blur are recognized warning signs for postoperative decompensation, and additional intraocular surgery may further stress the remaining endothelium [6]. Because no specular microscopy was obtained between the first operation and the IOL exchange, the proportion of the final 71–75% loss attributable to the first operation versus any additional loss caused by the exchange cannot be quantified. This case illustrates why objective endothelial assessment can be particularly valuable before elective re-intervention in an eye with unexplained postoperative edema.

The fellow eye provides a useful internal comparison, although it cannot serve as a formal control, as it was only a beginning cataract. It underwent FLACS also without documented intraoperative complications and subsequently showed only approximately 18–21% endothelial cell loss based on central measurements, with relatively preserved morphology despite regional variation in multipoint ECD measurements (Table 2). This comparison does not identify the mechanism of injury in the right eye, but the pronounced asymmetry makes a generalized bilateral endothelial disorder less likely and emphasizes that an apparently similar surgical approach can be followed by very different endothelial outcomes.

An additional clinically relevant observation is the longer-term course. Despite ECD measurements as low as 634 cells/mm^2^, the cornea did not progress to continuous decompensation requiring DMEK during approximately two years of follow-up. Corneal endothelial cells have limited proliferative capacity, but surviving cells can enlarge and redistribute to maintain pump function; consequently, ECD alone does not determine visual function at a single time point [5,6]. The earlier morning visual deterioration was compatible with marginal endothelial reserve, whereas the later improvement in visual acuity after Nd:YAG capsulotomy indicates that at least part of the subsequent visual limitation was attributable to posterior capsular opacification rather than progressive corneal failure. Continued serial endothelial monitoring remains appropriate because the functional reserve is nevertheless markedly reduced.

From a practical perspective, persistent or disproportionate corneal edema after FLACS should trigger a structured assessment before further intraocular procedures. Specular microscopy can quantify ECD and endothelial morphology, pachymetry can objectively document corneal swelling, and anterior-segment optical coherence tomography is useful when Descemet membrane detachment is suspected [5,12]. Early postoperative swelling itself may be informative, as the degree of day-1 corneal swelling has been shown to correlate with later permanent endothelial cell loss after phacoemulsification [7]. These examinations do not identify every mechanism, but together they can distinguish transient postoperative edema from a cornea with substantially reduced reserve and can guide the timing of subsequent intervention.

Future reports and prospective studies of rare severe endothelial injury after FLACS would be more informative if perioperative data were recorded in a standardized manner. Relevant variables include the laser platform and patient interface, number of docking attempts, ultrasound energy, fluidic parameters, ophthalmic viscosurgical device use, central corneal thickness, ECD, coefficient of variation, and hexagonality at defined postoperative time points. Such documentation could help determine whether severe outlier events reflect patient susceptibility, unrecognized mechanical or fluidic trauma, a particular procedural step, or the cumulative effect of more than one intraocular procedure.

This report has several limitations. As a single case, it cannot establish incidence or causality. No specular microscopy was obtained during the early postoperative period or immediately before the IOL exchange, preventing precise temporal attribution of the final endothelial cell loss. The reported docking event and other perioperative observations were patient-reported and were not documented as complications in the operative record. Detailed reconstruction of the exact mechanism is therefore not possible. Nevertheless, the presence of marked corneal edema and Descemet’s folds on postoperative day 1 documents clinically significant endothelial dysfunction before the IOL exchange, and the later serial measurements objectively demonstrate the severity and persistence of the endothelial deficit.

## 4. Conclusions

Severe endothelial cell loss may occur after apparently uncomplicated FLACS even when preoperative ECD is normal. In this case, marked corneal edema and Descemet’s folds were present on the first postoperative day and preceded a later IOL exchange; subsequent specular microscopy documented 71–75% endothelial cell loss. Persistent corneal edema or delayed visual recovery after cataract surgery should therefore prompt objective endothelial assessment, particularly before further elective intraocular intervention. The longer-term maintenance of corneal clarity and visual function despite markedly reduced ECD illustrates that functional compensation can persist despite limited reserve, but continued surveillance remains necessary. The precise mechanism of the initial endothelial injury remains uncertain, and more systematic perioperative documentation in severe outlier cases may help identify preventable risk factors.

## Figures and Tables

**Figure 1:**
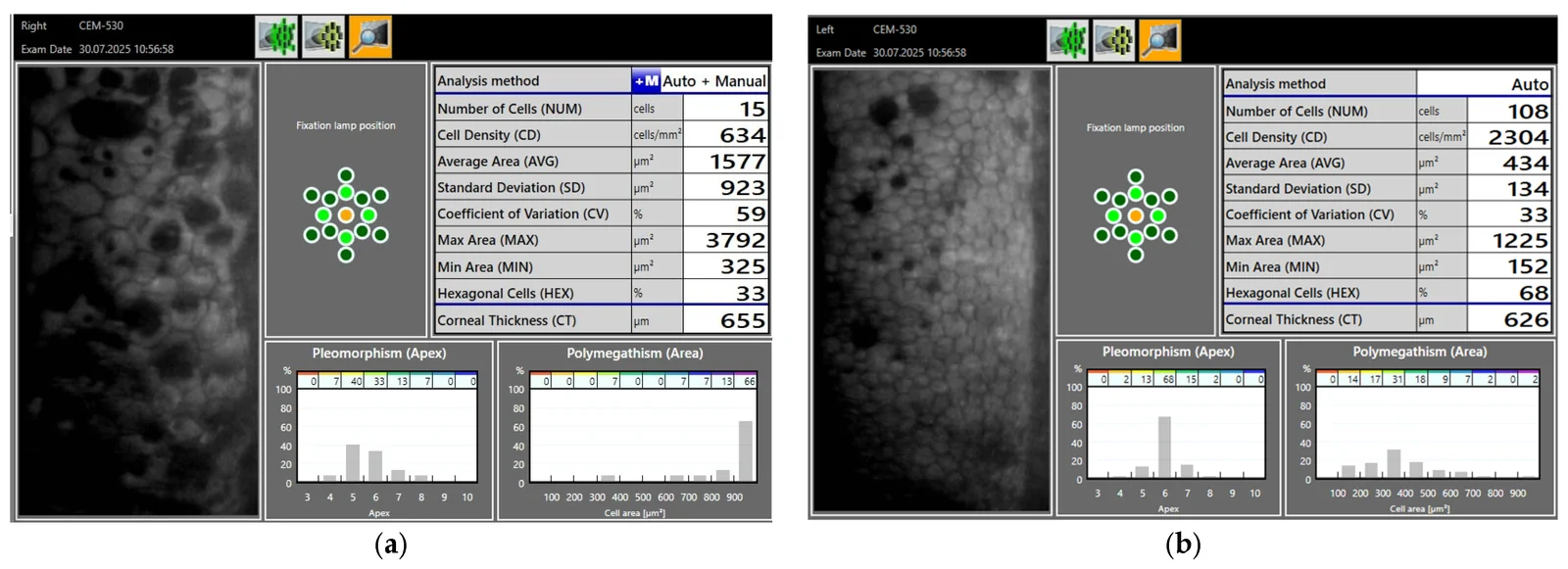
Corneal endothelial cell density and morphology on postoperative day 330 (30 July 2025). (**a**) Right eye after FLACS and subsequent IOL exchange: markedly reduced ECD (634 cells/mm^2^) with pronounced pleomorphism and polymegathism. (**b**) Left eye after FLACS: relatively preserved ECD (2304 cells/mm^2^) and morphology. Corneal thickness was 655 µm in the right eye and 626 µm in the left eye. The central right-eye value of 634 cells/mm^2^ was based on 15 identified cells in that individual field. However, this value was part of a five-field examination, with site-specific ECD values ranging from 634 to 957 cells/mm^2^ and 56 endothelial cells identified across the five fields in total.

**Table 1:** Timeline of clinical events and follow-up.

Timepoint	Event Description
Preoperative	Right eye: advanced nuclear cataract; left eye: early asymptomatic cataract; normal ECD in both eyes (right 2560 cells/mm^2^; left 2907 cells/mm^2^).
Day 0	Right-eye FLACS; implantation of monofocal Clareon™ Toric IOL (Alcon, Fort Worth, TX, USA); no intraoperative complication documented.
Day 1	Marked corneal edema with prominent Descemet’s folds; visual acuity 0.05–0.1; fundus not visible.
Days 6–13	Partial improvement of edema and Descemet’s folds; visual acuity 0.5–0.6; no specular microscopy performed.
Day 36	Persistent Descemet’s folds; visual acuity 0.63; corneal recovery still incomplete.
Day 41	IOL exchange: Clareon™ Toric explanted and AcrySof™ IQ Vivity™ Toric implanted (Alcon, Fort Worth, TX, USA); no endothelial cell count obtained before re-intervention.
Days 42–49	Transient postoperative corneal edema and Descemet’s folds after IOL exchange, followed by improvement; visual acuity 0.63 by day 49.
Day 65	Fellow-eye FLACS with AcrySof™ IQ Vivity™ EDOF IOL (Alcon, Fort Worth, TX, USA).
Day 176	ECD: right 743 cells/mm^2^ (71.0% loss); left 2309 cells/mm^2^ (20.6% loss).
Day 260	Clinical follow-up showed a clear cornea and IOP of 14 mmHg in both eyes; fine posterior capsular opacification was noted in the right eye. No endothelial cell measurement was performed.
Day 330	ECD: right 634 cells/mm^2^ (75.2% loss); left 2304 cells/mm^2^; marked pleomorphism and polymegathism in the right eye.
Day 428	Multipoint measurement showed persistently markedly reduced but regionally variable right-eye ECD; cornea remained clinically stable.
Day 644	Repeat multipoint ECD measurement confirmed persistently reduced and regionally variable right-eye ECD; the cornea remained clinically clear. Because of posterior capsular opacification, Nd:YAG laser capsulotomy was performed during the same visit. On the following day, decimal visual acuity was 1.0 in both eyes; DMEK was not considered necessary.

Abbreviations: DMEK: Descemet membrane endothelial keratoplasty; ECD: endothelial cell density; EDOF: extended depth of focus; FLACS: femtosecond laser-assisted cataract surgery; IOL: intraocular lens; IOP: intraocular pressure. Visual acuity values are reported in decimal notation.

**Table 2:** Serial corneal endothelial cell density measurements during follow-up.

Date	Eye	Central/Single-Site	0°	90°	180°	270°	ECD Loss vs. Preoperative Value
11 July 2024 (preoperative)	Right	2560	—	—	—	—	—
	Left	2907	—	—	—	—	—
26 February 2025	Right	743	—	—	—	—	71.0%
	Left	2309	—	—	—	—	20.6%
30 July 2025	Right	634	957	831	793	894	75.2%
	Left	2304	2686	2252	1865	2233	20.7%
5 November 2025	Right	968	771	691	964	786	62.2%
	Left	2332	2632	2164	2032	2342	19.8%
9 June 2026	Right	1046	1043	795	843	728	59.1%
	Left	2381	2704	2295	2061	2323	18.1%

## Data Availability

The clinical data presented in this case report are contained within the article. Additional clinical documents are not publicly available because they contain personal medical information and third-party records.

## Supplementary Materials

The following supporting information can be downloaded at: http://www.mdpi.com/article/10.3390/jcm15218465/s1, Figure S1: External photograph of the operated right eye obtained on postoperative day 3 (6 September 2024), showing a focal arcuate area of conjunctival hyperemia adjacent to the limbus. The photograph was taken by the patient. The origin of this finding and any relationship to the laser patient interface cannot be established retrospectively.
