# Supplementary material for: Severe Corneal Endothelial Cell Loss Following FLACS: A Case Report
Source: J Clin Med. 2026 Oct 30;15(21):8465. doi: 10.3390/jcm15218465 (PMC13763792; doi:10.3390/jcm15218465)
Supplement: Supplementary file 1 [file jcm-15-08465-s001.zip › jcm-4610498-supplementary.pdf]

Supplementary Figure S1

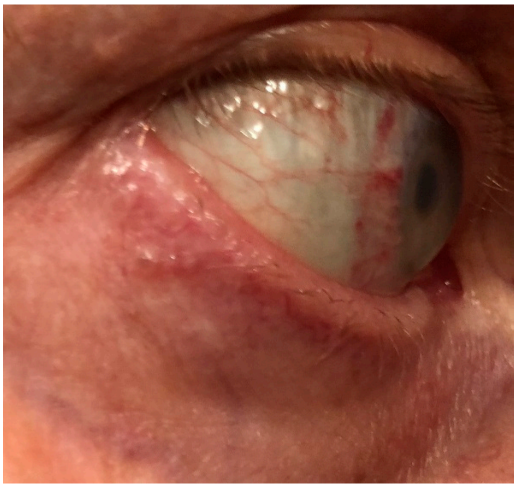

Figure S1. External photograph of the operated right eye obtained on postoperative day 3 (6 September 2024), showing a focal arcuate area of conjunctival hyperemia adjacent to the limbus. The photograph was taken by the patient. The origin of this finding and any relationship to the laser patient interface cannot be established retrospectively.
